# Limited Sustained Local Transmission of HIV-1 CRF01_AE in New South Wales, Australia

**DOI:** 10.3390/v11050482

**Published:** 2019-05-27

**Authors:** Francesca Di Giallonardo, Angie N. Pinto, Phillip Keen, Ansari Shaik, Alex Carrera, Hanan Salem, Barbara Telfer, Craig Cooper, Karen Price, Christine Selvey, Joanne Holden, Nadine Bachmann, Frederick J. Lee, Dominic E. Dwyer, Sebastián Duchêne, Edward C. Holmes, Andrew E. Grulich, Anthony D. Kelleher

**Affiliations:** 1 The Kirby Institute, The University of New South Wales, Sydney, New South Wales 2052, Australia; apinto@kirby.unsw.edu.au (A.N.P.); pkeen@kirby.unsw.edu.au (P.K.); ashaik@kirby.unsw.edu.au (A.S.); agrulich@kirby.unsw.edu.au (A.E.G.); akelleher@kirby.unsw.edu.au (A.D.K.); 2 Department of Infectious Diseases & Microbiology, Royal Prince Alfred Hospital, Camperdown, New South Wales 2050, Australia; 3 New South Wales State Reference Laboratory for HIV/AIDS, Darlinghurst, New South Wales 2010, Australia; alex.carrera@svha.org.au; 4 New South Wales Health Pathology, Royal Prince Alfred Hospital, Camperdown, New South Wales 2050, Australia; hanan.salem@health.nsw.gov.au (H.S.); frederick.lee@health.nsw.gov.au (F.J.L.); 5 Health Protection New South Wales, New South Wales Health, NSW, North Sydne, New South Wales 2060, Australia; barbara.telfer@health.nsw.gov.au (B.T.); christine.selvey@health.nsw.gov.au (C.S.); 6 Positive Life New South Wales, Surry Hills, New South Wales 2010, Australia; craigc@positivelife.org.au; 7 ACON Health Ltd., Surry Hills, New South Wales 2010, Australia; kprice@acon.org.au; 8 Centre for Population Health, New South Wales Ministry of Health, North Sydney, New South Wales 2059, Australia; jo.holden@health.nsw.gov.au; 9 Division of Infectious Diseases and Hospital Epidemiology, University Hospital Zurich, Institute of Medical Virology, University of Zurich, 8091 Zurich, Switzerland; nadine.bachmann2@usz.ch; 10 Department of Clinical Immunology & Allergy, Royal Prince Alfred Hospital, Camperdown, New South Wales 2050, Australia; 11 Sydney Medical School, University of Sydney, Sydney, New South Wales 2006, Australia; edward.holmes@sydney.edu.au; 12 New South Wales Health Pathology-Institute of Clinical Pathology and Medical Research, Westmead Hospital, Westmead, New South Wales 2145, Australia; dominic.dwyer@sydney.edu.au; 13 Department of Microbiology and Immunology, Peter Doherty Institute for Infection and Immunity, University of Melbourne, Melbourne, Victoria 3000, Australia; sebastian.duchene@unimelb.edu.au; 14 Marie Bashir Institute for Infectious Diseases and Biosecurity, Charles Perkins Centre, School of Life and Environmental Sciences, The University of Sydney, Sydney, New South Wales 2006, Australia

**Keywords:** HIV-1 CRF01_AE, transmission cluster, evolution, cluster growth, sub-epidemic

## Abstract

Australia’s response to the human immunodeficiency virus type 1 (HIV-1) pandemic led to effective control of HIV transmission and one of the world’s lowest HIV incidence rates—0.14%. Although there has been a recent decline in new HIV diagnoses in New South Wales (NSW), the most populous state in Australia, there has been a concomitant increase with non-B subtype infections, particularly for the HIV-1 circulating recombinant form CRF01_AE. This aforementioned CRF01_AE sampled in NSW, were combined with those sampled globally to identify NSW-specific viral clades. The population growth of these clades was assessed in two-year period intervals from 2009 to 2017. Overall, 109 NSW-specific clades were identified, most comprising pairs of sequences; however, five large clades comprising ≥10 sequences were also found. Forty-four clades grew over time with one or two sequences added to each in different two-year periods. Importantly, while 10 of these clades have seemingly discontinued, the remaining 34 were still active in 2016/2017. Seven such clades each comprised ≥10 sequences, and are representative of individual sub-epidemics in NSW. Thus, although the majority of new CRF01_AE infections were associated with small clades that rarely establish ongoing chains of local transmission, individual sub-epidemics are present and should be closely monitored.

## 1. Introduction

The human immunodeficiency virus type 1 (HIV-1) is still a major burden worldwide with approximately 36.9 million individuals infected with HIV globally (WHO). The Joint United Nations Programme on HIV/AIDS (UNAIDS) set the 90/90/90 targets with the aim that by 2020 90% of all people living with HIV-1 know their status, 90% of these will be receiving antiretroviral therapy, and 90% of these will have an undetectable plasma viral load [1]. The latter is of particular importance, as infected people that are virally suppressed do not develop the acquired immunodeficiency syndrome (AIDS) [2], and cannot transmit the virus further while on treatment [3]. Australia has an estimated HIV-1 prevalence of 0.14%, with approximately 1000 new infections annually [4]. New South Wales (NSW) is the most populous state in Australia, and has the highest HIV-1 burden, with approximately 10,100 people living with HIV-1 [5]. Importantly, NSW achieved the 90/90/90 targets in 2016 [5,6], and has experienced the first decline in new infections in ten years [7]. However, this decline was only observed in Australian-born men-who-have-sex-with-men (MSM), and an increase in new infections was observed in overseas-born MSM [6], indicating that there are differences in how different risk groups have been impacted by public health interventions.

HIV-1 circulates globally as different genetically defined subtypes, although the subtype prevalence varies across different countries; subtype C is most prevalent in Africa (and globally), the circulating recombinant form 01_AE (CRF01_AE) is most prevalent in Southeast Asia, and subtype B is most prevalent in the USA, Europe, and Australia [8]. This is of current importance because the HIV-1 epidemic in Australia is experiencing a shift in subtype incidence with a major increase in non-B infections, as an estimated 20% of all new infections in Australia were of non-B origin in 2006, and that increased to 38% in 2016 [9]. CRF01_AE is of particular interest for Australia due to the close proximity to Southeast Asia, with both travel and migration between these regions representing a conduit for HIV-1 importation [10]. Yet, little is known about the geographic origins and epidemiological characteristics of CRF01_AE in Australia.

To better understand the phylodynamics of CRF01_AE in NSW we (i) identified independent transmission clades, (ii) estimated clade growth over time, and (iii) assessed the impact of transmission clade growth on the NSW epidemic.

## 2. Materials and Methods

### 2.1. Ethics

This study was approved by the NSW Population and Health Services Research Ethics Committee and the ACON Research Ethics Review Committee (RERC) [AU RED Reference: HREC/15/CIHS/38, Cancer Institute NSW reference number: 2015/08/605]. The HIV/AIDS Legal Centre was consulted for legal advice on data anonymity.

### 2.2. HIV-1 Sequence Data

Protease and reverse transcriptase sequences sampled between 2004 and 2017 were obtained from the three laboratories performing HIV genotypic drug resistance testing for HIV-1 in NSW; St. Vincent’s HIV State Reference Laboratory, New South Wales Health Pathology-Royal Prince Alfred Hospital, and New South Wales Health Pathology Institute of Clinical Pathology and Medical Research (ICPMR). 

The final data set was created by the Center for Health Record Linkage (CHeReL) by linking the individual sequence identifiers obtained from the laboratories performing resistance testing to HIV notification case identifiers from the NSW HIV registry. Patient data always remained anonymized. The earliest available sequence for each patient was used for the analyses to avoid including multiple sequences from the same patient (*n* = 7000). HIV-1 subtype was determined using the REGA HIV-1 Subtyping Tool Version 3.0 [11]. 

### 2.3. Phylogenetic Analysis

A maximum likelihood tree including 83 HIV-1 reference sequences from the REGA tool and the 7000 NSW sequences was estimated using FastTreeMP v2.1.10 [12] via the CIPRES Science Gateway [13], implementing a GTR+Γ model of nucleotide substitution, and accounting for invariable sites, and NNI branch-swapping was implemented for topology search. Sequences forming a monophyletic CRF01_AE group (*n* = 758) were used for subsequent analyses. All HIV-1 sequences were retrieved from the Los Alamos HIV database (http://www.hiv.lanl.gov/) and used for comparison. NSW protease and reverse transcriptase nucleotide sequences were blasted against the global database, and the best 50 hits for each NSW sequence were retrieved from the global database [14]. Accordingly, a total of 2807 global sequences were used as background sequence data; three HIV-1 subtype G sequences (accession numbers DQ168576 and EU786670) were added as an outgroup to root the phylogeny (Table S1). Pairwise alignment was performed in Mafft, implementing the L-INS-I algorithm [15]. The sequences from the Los Alamos HIV database, as well as the sequences obtained from the three reference laboratories in NSW differ slightly in their length and genome position. As a consequence, the alignment was reduced to the nucleotide positions 2253–3384, based on the reference sequence HXB2 (accession number K03455.1). The alignment was visually inspected in Geneious 11.1.3 (https://www.geneious.com), and sequences that were too short were removed as well as codons associated with drug resistance mutations (according to reference sequence HXB2; accession number K03455.1) [16]. The final alignment comprised 3510 sequences of 1132 nucleotides in length, of which 702 were from NSW. Next, the NSW CRF01_AE sequences were split into four groups according to sampling year, with two new years of sampling added successively: (i) 2004–2009 (*n* = 149), (ii) 2004–2011 (*n* = 262), (iii) 2004–2013 (*n* = 387), (iv) 2004–2015 (*n* = 542), and (v) 2004–2017 (*n* = 702), the latter representing the complete data set. A maximum likelihood (ML) tree was estimated, employing FastTreeMP for each sub-data set, and using the same background data as described above. An approximate Shimodaira-Hasegawa test was implemented in PhyML v3.1. to test for node support [17,18]. Phylogenetic trees were visualized in FigTree v1.4.3. The high sampling density in this study and in potential subsequent studies enables the identification of complete transmission networks which would compromise patient privacy. Thus, a random subset of 10% of sequence data (*n* = 70) from the 702 CRF01_AE samples used in this study are available via NCBI under the accession numbers MK941065-MK941134 (Table S1).

### 2.4. Clade Identification

Clade identification was performed using R v3.4.3 [19], and a NSW-specific HIV-1 clade was defined as a node on the CRF01_AE phylogeny in which at least 80% of all tips were NSW sequences collected in this study [14,20,21]. ClusterPicker v1.2.5. [22] was used to identify potential active transmission clusters with that where characterized by a node support of 0.9 or higher and a genetic distance of 1.5% or lower [23,24].

## 3. Results and Discussion

### 3.1. Increase in non-B HIV-1 Subtype in NSW

**Figure 1:**
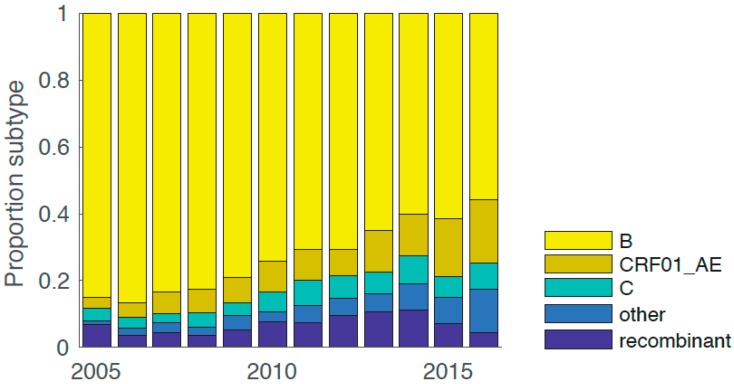
Changes in HIV-1 subtypes and circulating recombinant form 01_AE (CRF01_AE)_demographics over time. The proportion of subtypes is mapped across different years. Subtype B = yellow, subtype CRF01_AE = olive, subtype C = dark cyan, other non-recombinant subtypes = steel, and recombinants = blue.

Virus subtype was determined and changes in subtype incidence were measured over time for NSW HIV-1 sequences sampled between 2004–2017 (*n* = 7000). The proportion of non-B subtype sequences increased over time: Non-B subtypes comprised an average of 15% of all samples during 2004–2008, an average of 28% of all samples between 2009–2013, and 42% between 2014–2017 (Figure 1). Notably, CRF01_AE became the most prevalent non-B subtype and second most prevalent subtype overall in NSW, i.e., approximately 17% of all samples between 2014–2017 were CRF01_AE.

### 3.2. Identification of Large NSW-Specific Clades

**Figure 2:**
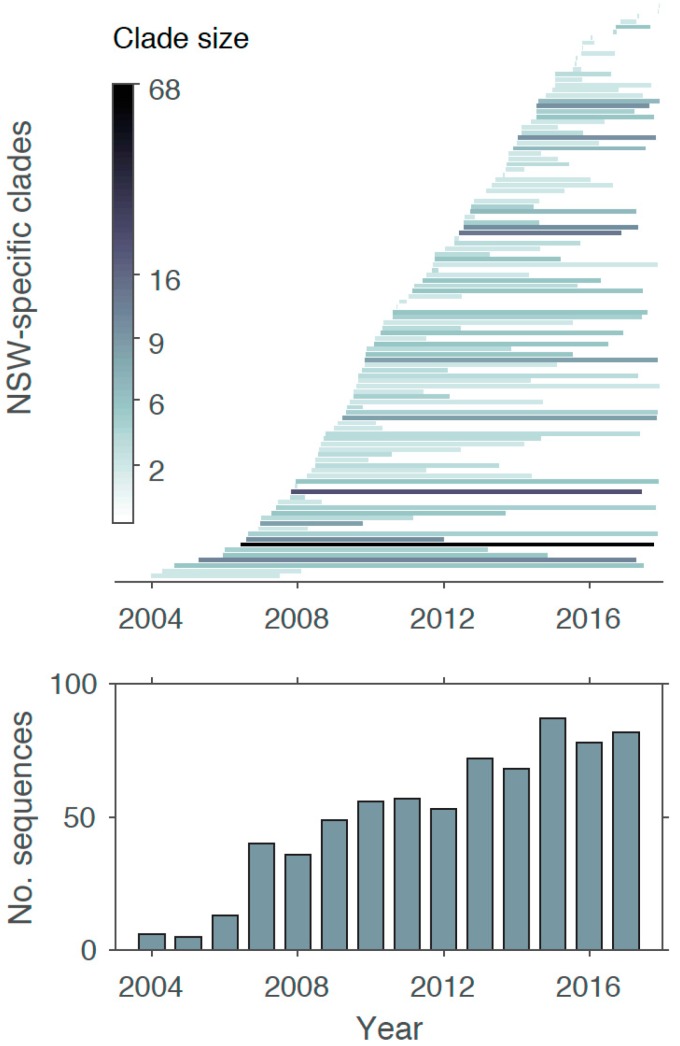
Sampling size changes over time. Number of sequences mapped across different years (bottom). New South Wales (NSW)-specific clade duration is shown by plotting the duration from the oldest to the most recent sequence sampled within a clade (top). Clades are colored according to clade size that represents the number of sequences within each clade.

Virus sequences were available from 2004 onwards. Although sequence numbers were low in the first three years, they increased steadily between 2007 and 2017, with an average of 62 sequences available for each year after 2007 (Figure 2). These data were combined with a background data set consisting of global sequences to identify NSW-specific independent clades (Figure S1). For the complete data set including all 702 NSW sequences, a total of 109 NSW-specific clades were identified, comprising 463 sequences, representing 66% of all NSW sequences in this study. A total of 50% (*n* = 54) of these clades were sequence pairs, with no evidence of additional onwards transmission. However, 355 sequences fell within 55 clades characterized by three or more sequences, and were distributed as follows: 19 clades contained three sequences, nine clades contained four sequences, 13 clades containing five sequences, three clades contained six and eight sequences each, two clades contained nine and ten sequences each, and four clades contained more than 10 sequences, i.e., 14, 16, 19, and 68 sequences, respectively. This clade size pattern is typical for HIV-1 phylogenies, with the majority of clades being small (two sequences), and only a handful of larger clades representing sub-epidemics [25,26]. This pattern also strongly supports multiple independent virus entries, i.e., individual sub-epidemics, of CRF01_AE in NSW, which arrived in NSW either via acquisition outside the state due to travel, or migration to NSW. 

We next mapped clade duration—that is, the time between the earliest and oldest sequences present in a clade—onto a time scale (Figure 2). Notably, while some small clades with two to four sequences were sampled within a short time frame, other small clades contain sequences sampled up to ten years apart. In fact, of the 100 clades identified, 51 spanned a time frame of three to 12 years, and 44 clades were sampled within one year. Thus, clade number and size appear to be growing over time.

Notably, the background data set contained 361 sequences sampled in Australia; however, only two of these formed a cluster with the NSW sequences, such that the contribution of inter-state transmission within Australia is seemingly limited, which is in concordance with a previous study showing that there is only limited clustering between sequences from different states [27].

### 3.3. Clade Growth is Slow over Time

**Figure 3:**
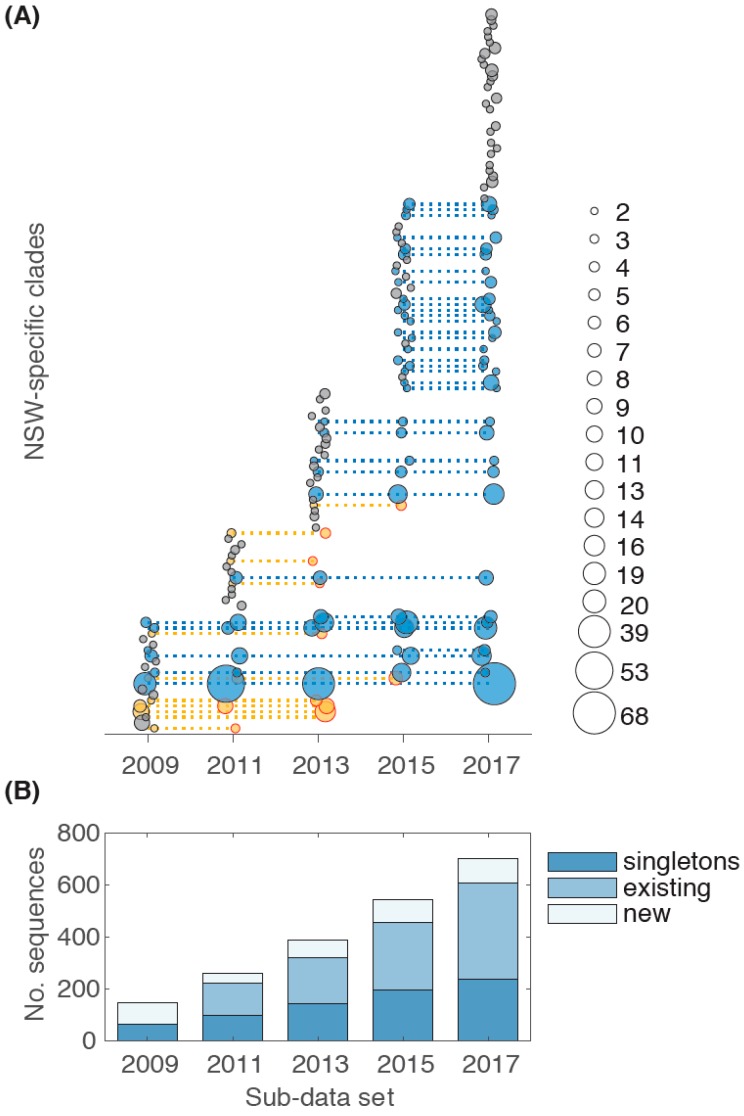
Discontinued and active ongoing NSW-specific clades. (**A**) Clade size for different clades is shown across the five sub-data sets. Discontinued clades are shown in orange with time period for the most recent sequence sampled shown with a red circle, sustained ongoing clades in blue, all other clades in black. Clade size for the analyzed time frame is indicated in the legend. (**B**) The number of singleton sequences is shown for the five different sub-data sets (dark blue). For each sub-data set new sequences belonging to an existing clade (blue) from the previous sub-data set, and new sequences forming a new clade (grey), are also shown. The x-axis defines the sequence sampling date grouped into the two-year time periods investigated.

To assess the rate of clade growth, NSW sequences were split into four different data subsets according to their year of sampling and combined with the global sequences. The first sub-data set comprised of 149 NSW sequences sampled between 2004–2009. A wider time range was chosen in this case to enable a solid starting point for clade identification. Two additional sampling years were added for each subsequent sub-data set. NSW-specific clades were identified for each sub-data set. Clades where the most recent sequence was sampled before 2016 were classified as temporarily discontinued, although these may include more recent undiagnosed cases (11% of all HIV-1 infected people living in Australia are thought to be undiagnosed [9]). Ten such discontinued clades were found (Figure 3A). Notably, 34 clades were still active in 2017 (ongoing clades) and have been steadily growing over time, with an addition of one or two sequences for each two-year period. This includes larger clades found comprising more than ten sequences, and including the largest clade with 68 sequences, all of which represent independent sub-epidemics. This is consistent with a previous study showing that clades grow slowly and irregularly [28]. 

Within the starting data set with sequences up to 2009, approximately 42% of the sequences represented singletons, and 58% were part of a clade (Figure 3B). Notably, this was the highest ratio of singleton sequences as the proportion of singleton sequences decreased over time in the following sub-data sets from 38% in the 2011 sub-data set to 24% in the 2017 data set. 

In contrast, the proportion of sequences forming a new cluster increased initially from 15% in the 2011 sub-data set to 17% in the 2013 sub-data set, but then decreased again to 14% in the complete data set (Figure 3B). This could simply be associated with an increase in the extent of sampling (HIV-1 testing). However, the number of new HIV notifications actually decreased in NSW from 332 in 2011, to 317 in 2016, and 313 notifications in 2017 [6]. Hence, while existing clades are growing more slowly, new clades are arising, such that public health interventions to stop virus transmission may be impacting on different populations unevenly. Indeed, epidemiological differences have been observed between HIV-1 positive individuals born in Australia, and those born overseas; for example a higher proportion of late diagnoses were observed in overseas born individuals [9]. However, from the 702 NSW CRF01_AE sequences used in this study, only 51% had available meta data including transmission risk and country of birth, so that no correlation between demographic factor and clade size and duration could be performed. 

### 3.4. Potential Active Transmission Networks 

Finally, potential direct transmission events were investigated by implementing a distance threshold of 1.5% and a node support of 99%. Sequences fulfilling these parameters form a transmission network, representing either direct virus transmission between individuals (i.e., a transmitter-recipient pair), or virus transmission to two recipients from the same transmitter. Sixteen singleton sequences had a high similarity to one or more of the global sequences, representing a potential transmitter-recipient pair between a sequence from overseas and a sequence sampled in NSW, although the directionality of transmission could not be established. In addition, four NSW-specific clades had a low genetic distance with one or more sequences from the global data set, indicating that transmission between sequences from different countries is not uncommon (Figure S2). 

Forty NSW-specific sequence pairs had a low genetic distance and high node support, indicative of a transmission network. Similarly, 13 networks with three sequences, four networks with five sequences, and one network with four, six, and eight sequences, respectively, were also identified. One of these has been active for at least six years, which is indicative of active clade growth, and which could be prevented by focused public health interventions. Notably, in one of the networks all three sequences share the drug resistance mutations K101E, Y181C and G190S, which can lead to reduced susceptibility to the non-nucleoside analog reverse transcriptase inhibitor drug class [16]. 

## 4. Conclusions

Our study has revealed the depth of active HIV-1 transmission for the CRF01_AE subtype in NSW, Australia. Importantly, the marked increase in CRF01_AE infections might be associated with an increase of small local transmission networks forming local sub-epidemics within the state. The slow growth in clade size and increase in clade number over time identified here highlights the likely effectiveness of interventions facilitating early diagnosis and contact-tracing.

## Supplementary Materials

The following are available online at https://www.mdpi.com/1999-4915/11/5/482/s1, Figure S1: Global phylogeny of CRF 01_AE; Figure S2: Potential active transmission networks; Table S1: Accession numbers.
