# Supplementary material for: Limited Sustained Local Transmission of HIV-1 CRF01_AE in New South Wales, Australia
Source: Viruses. 2019 May 27;11(5):482. doi: 10.3390/v11050482 (PMC6563510; doi:10.3390/v11050482)
Supplement: Supplementary file 1 [file viruses-11-00482-s001.zip › SupplementaryFigureLegends.docx]

**Supplementary Figure S1. Global phylogeny of CRF 01_AE.** Maximum likelihood tree of 702 NSW-specific sequences and 2,807 global sequences was estimated using the protease and reverse transcriptase region of 1,132 nucleotides. The tree was rooted using subtype G sequences as outgroup. The branch length indicates substitutions per site. Grey = global sequences; orange = NSW-specific sequences forming clades; blue = NSW-specific singleton sequences.

**Supplementary Figure S2. Potential active transmission networks.** Clades with a node support of 0.9 or higher and a genetic distance of 1.5% or lower were extracted from the ML tree seen in Figure S1. Sequence geographic origin and sampling year is indicated. Branches are coloured according to the network size. The branch length indicates substitutions per site.
