# Supplementary figures and images for: Limited Sustained Local Transmission of HIV-1 CRF01_AE in New South Wales, Australia
Source: Viruses. 2019 May 27;11(5):482. doi: 10.3390/v11050482 (PMC6563510; doi:10.3390/v11050482)

global data

clade

singleton

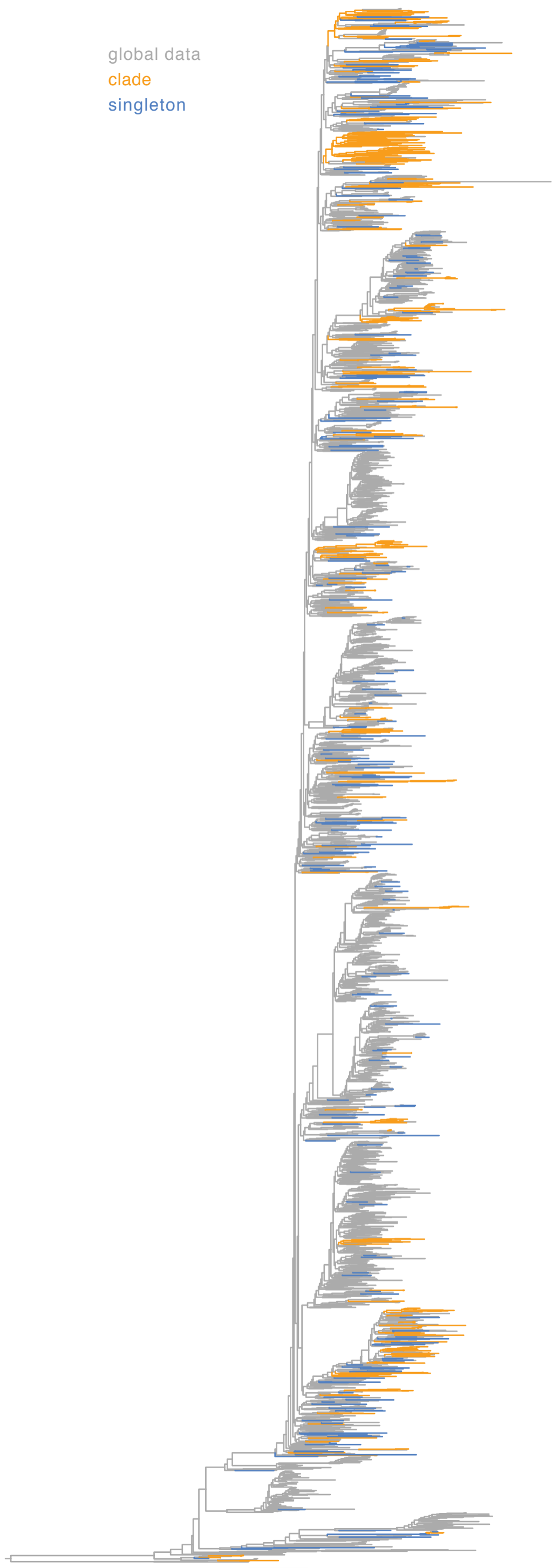

0.03

Supplement: Supplementary file 1 [file viruses-11-00482-s001.zip › DiGiallonardo_FigureS1.pdf]

## Transmission network size

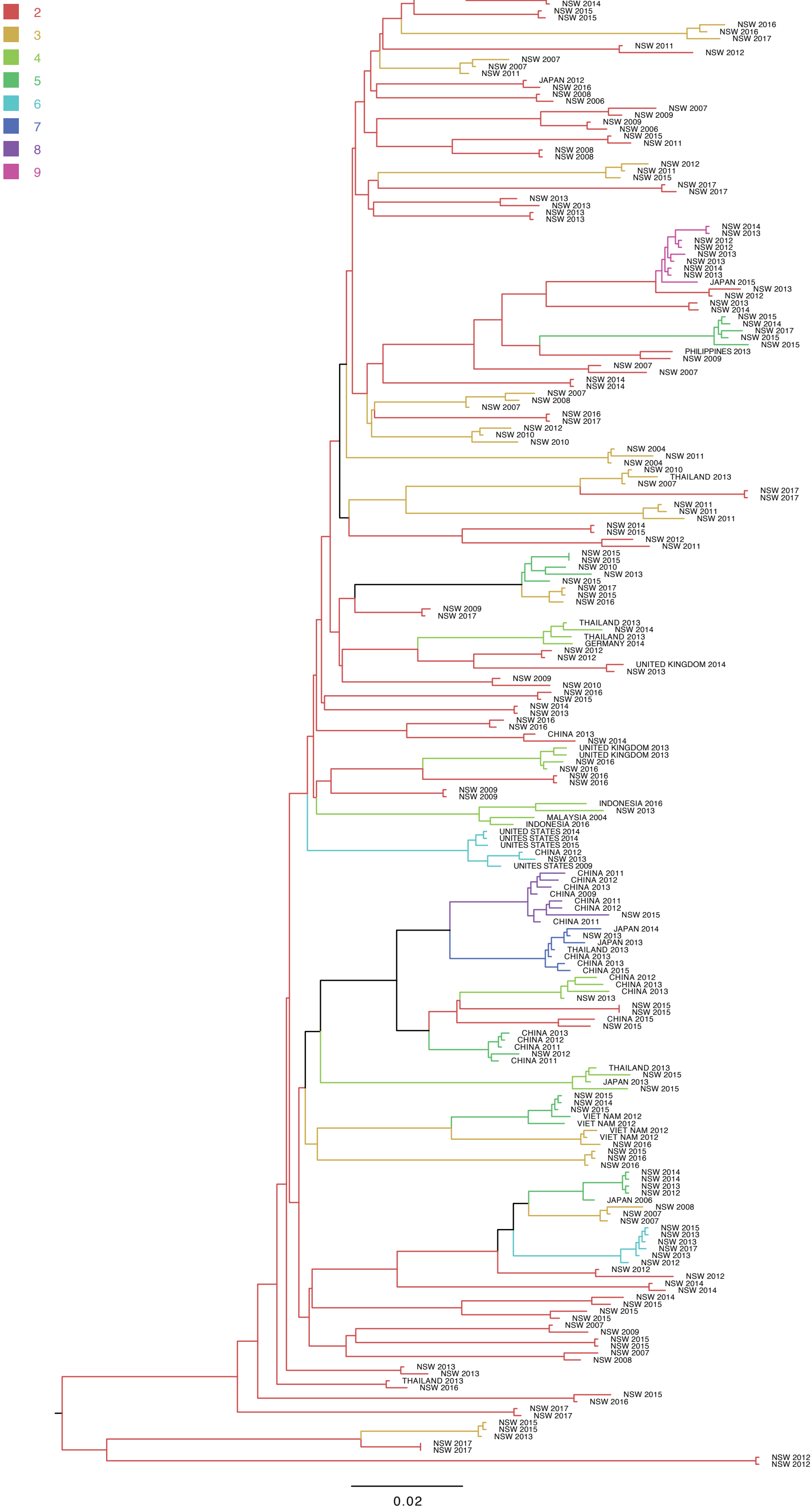

Supplement: Supplementary file 1 [file viruses-11-00482-s001.zip › DiGiallonardo_FigureS2.pdf]
